# Intention to Use an Electronic Community Health Information System Among Health Extension Workers in Rural Northwest Ethiopia: Cross-Sectional Study Using the Unified Theory of Acceptance and Use of Technology 2 Model

**DOI:** 10.2196/47081

**Published:** 2024-03-04

**Authors:** Tesfahun Hailemariam, Asmamaw Atnafu, Lemma Gezie, Jens Kaasbøll, Jorn Klein, Binyam Tilahun

**Affiliations:** 1 Department of Health Informatics, Institute of Public Health College of Medicine and Health Sciences University of Gondar Gondar Ethiopia; 2 Department of Health System and Policy, Institute of Public Health, College of Medicine and Health Sciences, University of Gondar Gondar Ethiopia; 3 Department of Epidemiology and Biostatistics, Institute of Public Health, College of Medicine and Health Sciences, University of Gondar Gondar Ethiopia; 4 Department of Informatics, University of Oslo Oslo Norway; 5 Department of Nursing and Health Sciences Campus Porsgrunn, University of South-Eastern Norway Porsgrunn Norway

**Keywords:** data capturing, data use, eCHIS, electronic community health information system, health extension worker, HEW, intention to use, service provision, Unified Theory of Acceptance and Use of Technology 2, UTAUT2 model

## Abstract

**Background:**

IT has brought remarkable change in bridging the digital gap in resource-constrained regions and advancing the health care system worldwide. Community-based information systems and mobile apps have been extensively developed and deployed to quantify and support health services delivered by community health workers. The success and failure of a digital health information system depends on whether and how it is used. Ethiopia is scaling up its electronic community health information system (eCHIS) to support the work of health extension workers (HEWs). For successful implementation, more evidence was required about the factors that may affect the willingness of HEWs to use the eCHIS.

**Objective:**

This study aimed to assess HEWs’ intentions to use the eCHIS for health data management and service provision.

**Methods:**

A cross-sectional study design was conducted among 456 HEWs in 6 pilot districts of the Central Gondar zone, Northwest Ethiopia. A Unified Theory of Acceptance and Use of Technology model was used to investigate HEWs’ intention to use the eCHIS. Data were cleaned, entered into Epi-data (version 4.02; EpiData Association), and exported to SPSS (version 26; IBM Corp) for analysis using the AMOS 23 Structural Equation Model. The statistical significance of dependent and independent variables in the model was reported using a 95% CI with a corresponding *P* value of <.05.

**Results:**

A total of 456 HEWs participated in the study, with a response rate of 99%. The mean age of the study participants was 28 (SD 4.8) years. Our study revealed that about 179 (39.3%; 95% CI 34.7%-43.9%) participants intended to use the eCHIS for community health data generation, use, and service provision. Effort expectancy (β=0.256; *P*=.007), self-expectancy (β=0.096; *P*=.04), social influence (β=0.203; *P*=.02), and hedonic motivation (β=0.217; *P*=.03) were significantly associated with HEWs’ intention to use the eCHIS.

**Conclusions:**

HEWs need to be computer literate and understand their role with the eCHIS. Ensuring that the system is easy and enjoyable for them to use is important for implementation and effective health data management.

## Introduction

Though IT has demonstrated remarkable promise in closing the digital divide in resource-constrained regions and advancing the health care system, there is a global shortage of health workers, which prevents at least half of the world’s population from receiving essential health services [1,2]. Training community health workers (CHWs) in low- and middle-income countries has been a recommended approach to closing the global shortage of health workers [2].

Ethiopia has been implementing the Health Extension Program (HEP) since 2003, comprising female health extension workers (HEWs) to improve the health status of its population [3]. Though various strategies were implemented, and substantial progress was observed in enhancing the community health information system (eCHIS), the performance of HEWs has remained low. The possible reasons for the low performance of HEWs are increased workload; lack of motivation; negligence; and skill gaps in health data production, use, and service provision [4-8].

Due to individual, organizational, and interpersonal level impediments, in most resource-constrained countries, particularly in sub-Saharan regions, health care data generated and used for decision-making are incomplete and inaccurate [9,10]. Likewise, quality health data generation and evidence-based decision-making practices are the remaining challenges for the health care system in Ethiopia [8,11,12].

The growing evidence shows that the penetration of mobile technology improves health service delivery and health outcomes across the world [13-19] and is becoming a solution to strengthen health care industries [8,20-22]. Previous studies in Ethiopia [23], Ghana [24], Uganda [25], South Dakota [26], Indonesia [27], Canada [28], Taiwan [29], South Korea [30], and Jordan [31] indicate that data collection using electronic systems may save time over manual data collection [32,33], and there is the potential to improve health care and the productivity of health staff. For example, digital health solutions may enable CHWs to generate quality health data [34], improve health care delivery [35], and help CHWs be more effective in their job at the community level [32,36].

The eCHIS is one of the evidence-based mobile platforms for CHWs in resource-constrained countries [37], which is an easily customizable mobile health (mHealth) platform for health workers to track and support their interactions with clients. It replaces the conventional practice of a CHW manually tracking their work and carrying large client data and documentation [37].

To tackle the challenges that existed with manual health data generation, use, and service provision, the Ethiopian Federal Ministry of Health has taken the initiative to digitize the existing paper-based Community Health Information System through the eCHIS and started piloting it in 6 districts of the Central Gondar zone, Northwest Ethiopia. The ultimate goal of its implementation is to improve the quality of health data production and service delivery at the community level by transforming the culture of information use by using tablet devices.

The first component of the eCHIS is the HEW component, which supports HEWs in family folder management and the provision of reproductive, maternal, newborn, and child health service delivery and follow-up. The second component is the health center referral component, which enables health center workers to confirm referrals and provide referral feedback to HEWs. The focal person component is the third component, which assists focal persons who are designated at the health center level to provide technical and programmatic support to the HEWs. Therefore, it enables HEWs to manage health post–level data and service provision, as it facilitates referral linkage of clients from health posts to health centers and vice versa.

Although using health system technology has expanded worldwide to leverage quality health data production and use, there is a paucity of evidence on users’ behavioral intention to use health system technology [38]. The intention to use a new system is how much a health care provider intends, plans, and predicts their future behavioral readiness to use health care technology [39]. Studies show that users’ behavioral intention is one of the significant factors of technology acceptance and use.

Hence, it is critical to evaluate the level of users’ intention to use IT before implementing it in the health care system [40-42], as it has a significant role in planning and designing effective implementation strategies for health care programs [43]. Moreover, identifying the level of intention to use the eCHIS for community health data production, use, and service provision and its influencing factors could help to be effective in the implementation and strengthening of the program. To the authors’ understanding, the level of HEWs’ intention to use the eCHIS for community health care data generation, use, and service provision has not been tested using the Unified Theory of Acceptance and Use of Technology 2 (UTAUT2) model.

The UTAUT2 model is one of the most mature IT models [44] that has emerged from 8 theoretical models that were primarily developed in psychology and sociology [45]. These include the Technology Acceptance Model, Theory of Planned Behavior, Combined Technology Acceptance Model and Theory of Planned Behavior, Theory of Reasoned Action, Motivational Model, Social Cognitive Theory, Model of PC Utilization, and Innovation Diffusion Theory [45,46].

The UTAUT2 has 3 broad types of integration of concepts. First, the integration was examined in new contexts, new users, and new cultural settings [46]. Second, the addition of new constructs increased the scope of dependent predictors [45]. Third, including independent predictors of the Unified Theory of Acceptance and Use of Technology (UTAUT) variables made comprehension easier [46]. Its extensive replications, applications, and integration extend the theoretical limits of technology adoption. Therefore, the addition of the 3 predictors (hedonic motivation, price value, and habit) to the previously existing 4 constructs in the original UTAUT model (performance expectancy, effort expectancy, social influence, and facilitating conditions) leveraged the adoption and use of technology (eCHIS in this case). This changes the existing relationships of constructs in the original UTAUT and introduces new relationships among constructs known in the UTAUT2.

We used the UTAUT2 constructs to determine HEWs’ behavioral intention to use the eCHIS [46], as UTAUT2 perspectives are applicable in the health system and the eCHIS is a form of health system technology. Understanding the intention of HEWs using the UTAUT2 model would give insights to health system leaders on how to digitize community health systems in local settings.

Therefore, this study aimed to investigate HEWs’ intention to use the eCHIS and its predicting factors using the UTAUT2 model among HEWs who had received familiarization training on the eCHIS in 6 pilot districts of Northwest Ethiopia.

Since the eCHIS is a form of health system technology, the relationships between UTAUT2 perspectives on accepting and using technology apply to the eCHIS, and the following hypotheses were speculated:

- Hypothesis 1: performance expectancy positively influences HEWs’ behavioral intention when using the eCHIS.
- Hypothesis 2: effort expectancy positively influences HEWs’ behavioral intention when using the eCHIS.
- Hypothesis 3: social influence positively influences HEWs’ behavioral intentions when using the eCHIS.
- Hypothesis 4: facilitating conditions positively influence HEWs’ behavioral intentions when using the eCHIS.
- Hypothesis 5: hedonic motivation positively influences HEWs’ behavioral intention in using the eCHIS.
- Hypothesis 6: self-efficacy positively influences HEWs’ behavioral intention when using the eCHIS.
- Hypothesis 7: habit positively influences HEWs’ behavioral intention when using the eCHIS.

In this study, price value was not included in this model because HEWs, the participants in this study, were not directly involved in purchasing the system. Furthermore, the model was not tested on behavioral intention to use the eCHIS among HEWs in Ethiopia.

## Methods

### Study Design, Period, and Setting

A cross-sectional study design was conducted from January to February 2021 in the Central Gondar zone, Northwest Ethiopia. The Central Gondar zone has 15 districts, of which 6 districts (Wogera, Mirab Dembia, Misrak Dembia, Enfranz, Takusa, and Belesa) were selected as pilot districts for eCHIS implementation in the zone. The estimated total population of the zone was 2,288,440. The zone has a total of 75 health centers and 404 health posts, and there were 897 HEWs (59 urban and 848 rural) during the study period (Central Gondar Zone Health Bureau report, unpublished data, 2020).

### Population and Participants of the Study

The source population of the study was HEWs at the primary health care unit level. The study participants were HEWs who were in the pilot districts of the Central Gondar zone and had received initial training for eCHIS implementation. The intervention was skill-oriented training for the implementers of mobile-based community health information system applications based on the training manual prepared by the Ministry of Health, and the training was provided for 1 week by trainers from the regional health bureau and the Ministry of Health. Following the training, each *woreda* (district) led household registration, tablet usage guideline provision, technical support and mentoring, and periodical communications for 1 year.

### Provision of Mentorship and Technical Assistance

The University of Gondar assigned three supporting team members who provided technical assistance for implementers with a local mentor every 2 weeks throughout the intervention period. In addition, 1 health information technician (a local mentor) was assigned to provide mentorship and solve eCHIS-related problems during implementation.

### Sample Size and Sampling Procedures

The initial sample size was calculated using a single population proportion formula, considering the following assumptions: 50% proportion of intention to use the eCHIS, as there was limited evidence in the area; 95% confidence level for estimations; and 5% margin of error. Using these inputs, the initial sample size was estimated at 385. Considering a 10% nonresponse rate, the final sample size was 422. In the pilot districts, however, the total number of HEWs was 460. Therefore, as the initially determined sample size was closer to the population size, it was planned to include all eligible HEWs in the study.

### Study Variables and Measurement

The dependent variable was the intention to use the eCHIS for health data generation and service provision. Based on the UTAUT2, 8 constructs with a 5-point Likert scale were used to assess the intention to use the eCHIS and were considered potential predictors of the study [46].

1. Performance expectancy: the extent to which people believe that using a new technology can improve their job performance [47].
2. Effort expectancy: the degree of ease of use associated with the usage of a new technology [46].
3. Social influence: the degree of importance others recognize in using a new system [45].
4. Facilitating conditions: the degree to which a person perceives that an organization and a technical infrastructure exist to support the intention of people to use technology [45].
5. Hedonic motivation: the motivation to do something due to internal satisfaction [48].
6. Habit: the degree to which users perform the usage of technologies behaviors automatically because of learning [46,49].
7. Self-efficacy: judgment of one’s ability to use technology to accomplish a particular job or task [45].
8. Behavioral intention: the degree to which a person has formulated conscious plans to perform or not perform some specified future behaviors [50].

### Data Collection Tools and Procedures

Data collection tools were adapted from the source instrument used in the UTAUT2 model [46] in the context of the eCHIS to enhance comprehension by the respondents. The items in the constructs were performance expectancy (4 items), effort expectancy (4 items), social influence (3 items), facilitating condition (4 items), hedonic motivation (3 items), self-expectancy (4 items), habit (4 items), and intention to use (3 items). The source language of the instrument was translated forward into the local language of Amharic, and a backward translation was done to ensure the consistency of the tool. Experts with health management information system backgrounds were invited to review the relevance of each question in the instrument. The experts reviewed the instrument and checked its content and face validity, and the instrument was refined according to the comments given. A pretest was conducted on 5% of the study participants before actual data collection was started, and the tool was refined based on the pretest results. A total of 4 data collectors and 2 supervisors were recruited and trained on the purpose, tools, and procedures of the study. Self-administrated questionnaires were used to collect data from HEWs with the assistance of data collectors and supervisors. The data collection period was from January 28 to February 13, 2021, after 2 weeks of eCHIS familiarization training had been given.

### Data Management and Analysis

The data were entered into Epi-data (version 4.02; EpiData Association) and exported to SPSS (version 26; IBM Corp) for descriptive statistics such as frequency, cross-tabulations, and univariate analysis of sociodemographic and model constructs. Simple and multiple structural equation models were carried out using the AMOS 23 Structural Equation Model in order to test the relationship between observed and latent variables and identify the predicting variables of the intention to use the eCHIS. During analysis, we applied a parceling technique to increase model efficiency [51]. The subset-item-parcel approach was used in order to aggregate items into several parcels and use them as indicators of the target construct [52]. Accordingly, we created 2 parcels for each factor of target latent constructs (such as performance expectancy, effort expectancy, facilitator conditions, self-expectancy, and habit) by aggregating randomly grouped items within each scale [53]. The remaining 3 latent target constructs with 3 indicators per construct, such as social influence, hedonic motivation, and intention to use, remained as they existed in the original UTAUT2 model [46].

The overall model’s fitness was measured and assessed using the goodness of fit indices such as chi-square ratio (<3), the goodness of fit index (>0.9), adjusted goodness of fit index (>0.8), normal fit index (>0.9), comparative fit index (>0.9), Tucker-Lewis index (>0.9), and root mean square error of approximation (<0.08). For the structural equation model, standardized path coefficients of the regression weight values were used to estimate the path coefficients of the dependent and independent variables. Standardized coefficients are not dependent on the scales as they vary from –1 to 1, where 0 indicates no relationship, 1 indicates a strong positive relationship, and –1 indicates a strong negative relationship. A critical ratio (regression weight or standard error) was used to evaluate whether the constructs had a significant relationship. The absolute value of a critical ratio greater than 1.96 is an indication of the significance of the path coefficients. In this study, the CI and its *P* value were calculated using bootstrapping, and the statistical significance of dependent and independent variables in the model was reported using a 95% CI with a corresponding *P* value of <.05 (Table 1). The square multiple correlation (*R*^2^) was used to report the proportion of variance so that the intention to use the eCHIS could be explained by the model.

**Table 1:** Structural equation modeling fitness for intention to use electronic community health information system among health extension workers in Northwest Ethiopia, 2021.

Fit indices	Threshold value	Authors	Results obtained	Conclusion
Chi-square	<3	Bentler [54] (1990)	2.67	Accepted
Goodness-of-fit index	>0.9	Chau [55] (1997)	0.92	Accepted
Adjusted goodness-of-fit index	>0.8	Chau [55] (1997)	0.88	Accepted
Comparative fit index	>0.9	Bentler [54] (1990)	0.97	Accepted
Root mean square error of approximation	<0.05	Browne and Cudeck [56] (1993)	0.08	Accepted
Normed fit index	>0.9	Bentler and Bonett [57] (1980)	0.95	Accepted

### Reliability and Validity of the Research

Regarding the reliability and validity of the study, Cronbach α reliability coefficients were computed to determine the internal consistency of the constructs. Cronbach α of .7 or above indicates high reliability; between .5 and .7 indicates moderate reliability; and less than .5 indicates low reliability. We have used 4-item Likert questions to assess the reliability of the constructs. Accordingly, the reliability of the constructs assessed by 3-item questions as follows: performance expectancy (α=.92), effort expectancy (α=.87), facilitating condition (α=.75), self-expectancy (α=.88), habit (α=.84), social influence (α=.78), hedonic motivation (α=.90), and intention to use eCHIS (Table 2). In this study, the magnitude of intention to use the eCHIS was assessed by a 3-item Likert question with a reliability test of Cronbach α=.93.

**Table 2:** Reliability of the constructs on intention to use the electronic community health information system (eCHIS) among health extension workers in Northwest Ethiopia, 2021.

Constructs	Sample size	Number of items	Cronbach α
Performance expectancy	456	4	.92
Effort expectancy	456	4	.87
Social influence	456	3	.78
Facilitating conditions	456	4	.75
Hedonic motivation	456	3	.90
Self-expectancy	456	4	.88
Habit	456	4	.84
Intention to use the eCHIS system	456	3	.93

### Ethical Considerations

Study approval and ethical clearance were obtained from the University of Gondar’s ethical review board (R.NO. V/P/RCS/05/2020) and a support letter from the ethical review committee of the Amhara Regional Health Bureau Research and Technology transfer office. Study permission was sought at all levels of governmental administration systems including health offices and health facilities. Written consent was obtained, and participants were informed about the objective, importance of the study, procedure and duration, risk and discomfort, benefits of participating in the study, confidentiality, and the right to refuse or withdraw during data collection. To ensure confidentiality, their names and other personal identifiers were not registered. Participants were not compensated for study participation. We confirm that the provided ethics approval documentation covers the study presented in this manuscript.

## Results

### Sociodemographic and Other Characteristics of the Study Participants

A total of 456 HEWs participated in the study, with a response rate of 99%. The mean age of the study participants was 28 (SD 4.8) years. More than two-thirds (n=314, 68.9%) of the study participants had work experience of more than 5 years. About half of the participants (n=232, 50.9%) were level 4 (10+4) in their educational status, and the majority of the respondents (n=307, 67.3%) were married. The number of HEWs who had difficulties recharging mobile phones was 307 (67.3%). Our study found that 147 (32.2%) HEWs used Microsoft applications daily , 331 (72.6%) had experience using mobile phones for more than 5 years and above, and 421 (92.3%) had informal mobile phone usage practices or were using personal mobile for health post–related activities (Table 3).

According to the findings of this study, 122 (26.8%), 132 (28.9%), and 162 (35.5%) HEWs strongly agreed to intend, predict, and plan to use the eCHIS, respectively (Table 4).

**Table 3:** Sociodemographic and informal phone use characteristics of the study participants in Northwest Ethiopia, 2021.

Variables and categories		Values (N=456), n (%)	
**Age group (years)**			
	<24	104 (22.8)	
	25-34	295 (64.7)	
	≥35	57 (12.5)	
**Marital status**			
	Married	307 (67.3)	
	Single	118 (25.9)	
	Divorced	26 (5.7)	
	Widow	5 (1.1)	
**Work experience (years)**			
	0-2	55 (12.1)	
	3-5	87 (19.1)	
	>5	314 (68.9)	
**Level of education**			
	Level I	5 (1.1)	
	Level II	12 (2.6)	
	Level III	196 (43)	
	Level IV	232 (50.9)	
	Others^a^	11 (2.4)	
**Difficulty with battery recharging**			
	Yes	307 (67.3)	
	No	149 (32.7)	
**Using Microsoft applications for work and daily life**			
	Yes	147 (32.2)	
	No	309 (67.8)	
**Do you use personal mobile phone for health post–related activities?**			
	Yes	421 (92.3)	
	No	35 (7.7)	
**For how long you have used mobile phone (years)?**			
	0-5	125 (27.4)	
	>5	331 (72.6)	

^a^Health extension worker with additional diploma, BSc degree, or both.

**Table 4:** Health extension workers’ intention to use the electronic community health information system (eCHIS) in Northwest Ethiopia, 2021 (N=456).

Items	Strongly disagree, n (%)	Disagree, n (%)	Neutral, n (%)	Agree, n (%)	Strongly agree, n (%)
I intend to use the eCHIS system in the future	7 (1.5)	12 (2.6)	12 (2.6)	303 (66.4)	122 (26.8)
I predict I will use the eCHIS system in the future	5 (1.1)	13 (2.9)	9 (2)	297 (65.1)	132 (28.9)
I plan to use the eCHIS system in the future	4 (0.9)	11 (2.4)	15 (3.3)	264 (57.9)	162 (35.5)

### Mean Score of All Predictors and Intention to Use the eCHIS Using the UTAUT2 Model

The mean scores of performance expectancy, effort expectancy, facilitating condition, self-expectancy, and habit with 4-item Likert questions were 17.09 (SD 2.58), 16.22 (SD 2.41), 12.07 (SD 3.46), 13.94 (SD 3.62), and 14.75 (SD 3.14), respectively. On the other hand, social influence, hedonic motivation, and intention to use the eCHIS with 3-item Likert questions had a mean score of 11.63 (SD 2.32), 12.23 (SD 2.01), and 12.57 (SD 2.00), respectively.

Our study revealed that 179 (39.3%; 95% CI 34.7%-43.9%) participants who had the intention to use the eCHIS for community health data generation, use, and service provision had scored above the mean. The mean score of the intention to use the eCHIS was 12.57 (SD 2.00). The maximum score of intention to use the eCHIS was 15, while the minimum score was 3.

### Simple Structural Equation Model Analysis

**Figure 1:**
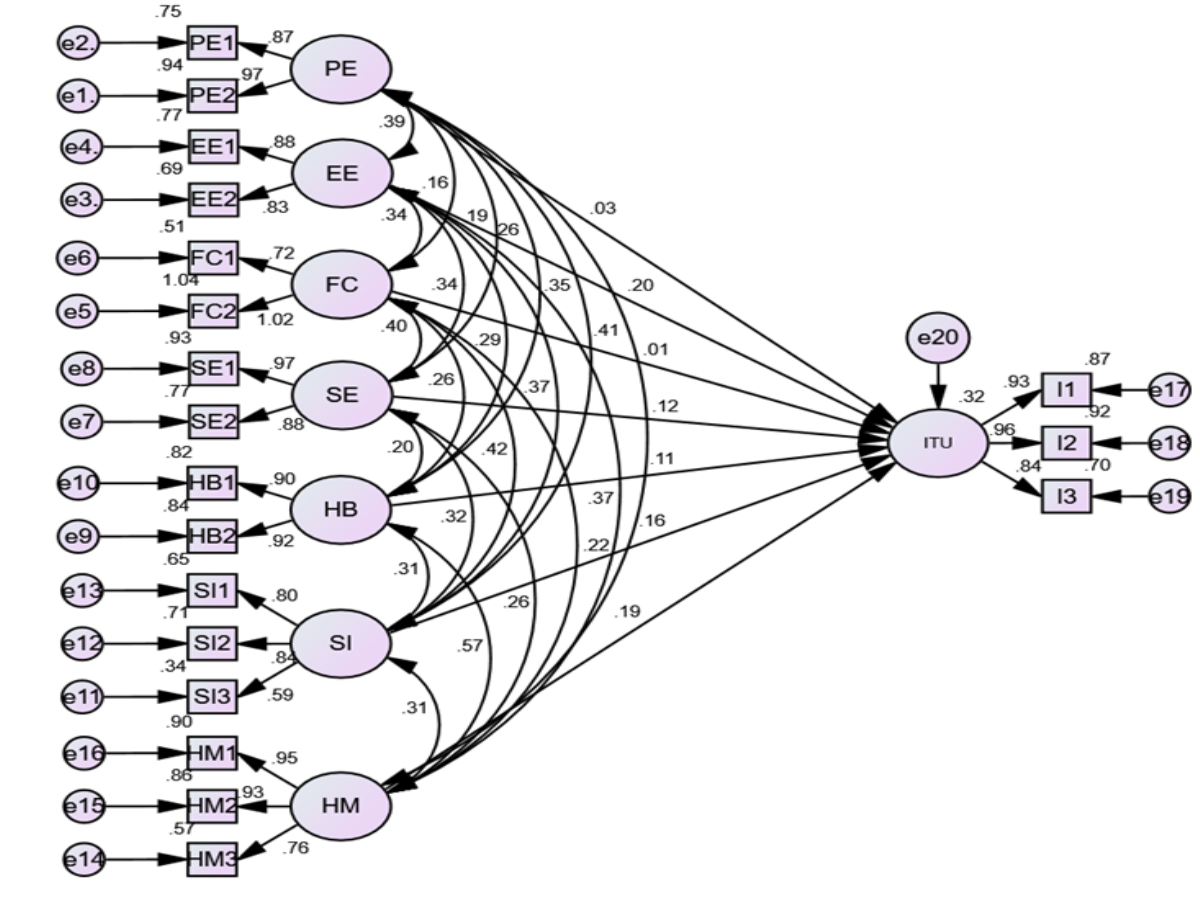
Predictors and intention to use the electronic community health information system among health extension workers at Central Gonda zone, Northwest Ethiopia, 2021. EE: effort expectancy; FC: facilitating condition; HB: Habit; HM: hedonic motivation; ITU: intention to use; PE: performance expectance; SE: self-expectancy; SI: social influence.

The variance in the dependent variable explained by the independent variables was interpreted using square multiple correlation (*R*^2^). The overall *R*^2^ of the intention to use the eCHIS is found to be 32%, the variance that was explained by the independent variables in the model. The bootstrap method with a 95% bias-corrected CI was applied to investigate the significance of path coefficients and factors predicting the model. The predictors with *P*<.20 in the simple structural equation model were considered candidate variables for multiple structural equation model analysis. Due to its undependability to scale, we used a standardized beta coefficient to interpret the influence of predictors on the intention to use the eCHIS. A 95% CI with *P*<.05 was considered to declare an association between dependent and independent variables. The study indicated that effort expectancy has the highest direct effect on HEWs’ intention to use the eCHIS, followed by hedonic motivation. The remaining model constructs that have a direct influence on predicting intention to use the eCHIS are social influence and self-expectancy. The structural equation model predicted, with the path coefficients and *R*^2^, is represented in Figure 1, and the path coefficients and *P* value found from the depicted model are presented in the *Results* section. Moreover, the absolute value of the critical ratio of effort expectancy (3.701), self-expectancy (2.468), social influence (2.782), and hedonic motivation (3.311) indicated that predictors had a significant influence on HEWs’ intention to use the eCHIS. Overall, 32% of the variance with respect to intention to use the eCHIS was reasonably explained by the predictors in the model.

Effort expectancy (the extent to which people believe that using the eCHIS can improve their effort) has a positive influence on HEWs’ behavioral intention (β=.256; *P*=.007). Similarly, self-efficacy and social influence had a positive influence on HEWs’ behavioral intention (β=.096; *P*=.04), and (β=.203; *P*=.02), respectively. Likewise, hedonic motivation to use eCHIS due to internal satisfaction was found to be (β=0.217; *P*=.03) and had a significant effect on intention to use the eCHIS. Facilitating conditions (β=0.005; *P*=.92), habit (β=0.103; *P*=.07), and performance expectancy (β=0.034; *P*=.61) had no significant influence on intention to use the eCHIS (Table 5).

**Table 5:** Multiple structural equations modeling association between predictors and intention to use the electronic community health information system among health extension workers in Northwest Ethiopia, 2021.

Hypothesis	Estimate	95% CI	*P* value	Decision
PE^a^ ⇒ IU^b^	0.034	–0.91 to 0.190	.61	Not supported
EE^c^ ⇒ IU	0.256	0.060 to 0.503	.007	Supported
FC^d^ ⇒ IU	0.005	–0.060 to 0.079	.92	Not supported
SE^e^ ⇒ IU	0.096	0.004 to 0.191	.04	Supported
HB^f^ ⇒ IU	0.103	–0.008 to 0.235	.07	Not supported
SI^g^ ⇒ IU	0.203	0.039 to 0.390	.02	Supported
HM^h^ ⇒ IU	0.217	0.022 to 0.416	.03	Supported

^a^PE: performance expectance.

^b^IU: intention to use.

^c^EE: effort expectancy.

^d^FC: facilitating condition.

^e^SE: self-expectancy.

^f^HB: habit.

^g^SI: social influence.

^h^HM: hedonic motivation.

## Discussion

### Principal Findings

In this study, nearly 2 out of 5 HEWs had an intention to use the eCHIS for community health data generation, use, and service provision. Effort expectancy, self-expectancy, social influence, and hedonic motivation were statistically significant predictors of intention to use the eCHIS. The intention to use the eCHIS by HEWs could be associated with the fact that using the eCHIS is not difficult to understand. It saves time and reduces the amount of effort required to complete health-related tasks [58,59]. Furthermore, it simplifies activities and helps them access data easily. The other could be people around them who have the ability to influence their intention to use the system [58]. For example, HEWs’ activities should be monitored and evaluated by health system leaders. If they give them more attention, they will be encouraged to use the system. The other could be previous exposure to using informal phone for health system activities, such as reminding clients about their health care appointments and facilitating referral linkage between health centers and health posts, as mHealth enhances communication between health workers and clients [58]. Moreover, using the eCHIS creates a conducive environment for HEWs since their usual data handling approach is exhaustive and takes much time to execute activities at the health post level, and using the eCHIS not only helps them to save their time but also creates motivation to do their job at health post level [58,60].

Regarding factors associated with intention to use the eCHIS, effort expectancy had a positive influence on the intention to use the eCHIS among HEWs. This finding was in accordance with a study conducted in Ethiopia [61], Kenya [61], the United States [62], and Portugal [63] and had a positively significant association with the intention of health care providers to use technology. A possible explanation could be the fact that the less effort the user devotes to using the system, the more likely he or she is to continue to use it. A study in this regard showed that individuals often want to face a system that is easy to use [64]. HEWs might perceive that the eCHIS could help them to do their job aids shortly with less strain and increased work efficiency [65], as using the eCHIS would simplify the tasks they are expected to deliver at health post level. A review in this regard showed that using digital tools simplifies work and helps to access data easily [59]. Furthermore, studies indicate that digital health solutions reduce workload and improve work performance [24,25], reduce errors [34], create motivation and learning opportunities [66], promote health care appointment [67], and are easy to use and improve work efficiency [59]. Using the eCHIS could reduce the workload of HEWs since manual data management practice at health post level is exhaustive and takes much time to collect data and conduct routine activities [59]. Moreover, the referral linkage integrated into the eCHIS, including HEWs, midwives, and focal persons, will harmonize HEWs’ activity flow from health posts to health centers and vice versa. Furthermore, using mHealth motivates CHWs and enables them to perform multiple tasks quickly, reducing efforts and improving performance [60].

The intention to use the eCHIS among HEWs who perceived people around them could influence their behavioral intention was positively associated. The current finding corroborates studies conducted among health care providers using the UTAUT2 model in Ethiopia [61], Morocco [68], Taiwan [62], South Korea [30], and the United States [69], showing that social influence significantly predicted health care providers’ intention to use technology. The possible explanation could be that HEWs might perceive peer pressure from health care staff at the *woreda* and facility levels toward using the eCHIS, which could positively influence their intention to use the eCHIS. The other justification could be the fact that HEWs might get trust from the community for the job aids or activities they are expected to deliver. Hence, health system staff need to understand that peer influence has a positive effect on using a new system. Moreover, making people aware of a new system at the *woreda* and facility levels in general and at the kebele leaders, women’s development army, and voluntary service providers’ levels, in particular, could influence HEWs’ behavioral intention to use the eCHIS. A study in this regard showed that the more health workers connected to colleagues, the more they improved the use of digital tools and the quality of care [58].

Our study revealed that the magnitude of intention to use the eCHIS among HEWs who had self-expectancy was positively correlated. The findings of past studies in Ethiopia [61], Malaysia [70], Taiwan [71], and Iran [72] showed that digital literacy was correlated with the intention to use technology in health care industries. The possible reason might be that those who had self-expectancy could not face difficulty in adapting the emerging technology to community-level data management and service provision. The current evidence in the feasibility and effectiveness study on digital health indicated that the level of computer literacy had influenced digital health implementation among CHWs [73]. A possible explanation might be the fact that informal mobile phone usage practices of CHWs for health post–related activities could influence behavioral intention to use the eCHIS. A study indicated that in many different settings, CHWs use their personal phone informally for community-based activities so as to fill the gaps in the health care system [74].

Our study revealed that there is a significant association between intention to use the eCHIS and hedonic motivation or perceived enjoyment from using the eCHIS for community health data generation, use, and service provision. A possible explanation could be the fact that using a new system instead of the usual approach to manage community-level data and service provision may create intrinsic motivation for HEWs to obtain fun or pleasure. A study showed that motivation is an important construct for eHealth users, and it could even be a sufficient reason to adopt newly emerging technology in a contextual environment [75]. In addition, using eHealth technology to deal with community health data generation, use, and service provision may be an enjoyable process and will have a positive influence on the behavioral intentions of the users [72].

HEWs were optimistic about using the eCHIS because it could be related to the production of quality health data, ease of data management, reduced errors and false reports, data protection, and increased accessibility. A study also indicated that using digital tools could enhance the productivity of CHWs [76]. Community health digitization using mobile apps support the services delivered by CHWs [77]. Furthermore, studies show that the digitization of health care data has promising results in improving both health care and health outcomes [13-19] and improving health staff productivity and work efficiency [65]. In our study, the level of users’ optimistic perceptions of using the eCHIS could be an advantage in implementing the intervention, as compared to the existing approach [78]. Studies showed that digital health solutions enable CHWs to generate quality health data [34], improve health care delivery [35], and help them to be more effective in their job aids [32,36]. Moreover, digital tools could help them follow the correct order or the required service elements that clients should receive when providing services. It also enables them to communicate with clients in a better way as compared to manual communication since the tool has prespecified data elements that should be asked by CHWs during service delivery [58].

Likewise, it creates enjoyment among users [66] and benefits them by keeping data safe from human and natural factors that could damage the data. In addition, enjoyment could emanate due to the fact that using digital tools can improve data capturing, storing, and reporting of more items that could be more time-consuming during manual data handling and reduce the motivation of health workers to keep data recording [59]. Even though HEWs are optimistic to use the eCHIS, lack of adequate resources for eCHIS implementation at the implementation district could hinder its successful implementation, and therefore resource availability is vital to be effective in community health digitization. Studies show that challenges during digital health solutions implementation, such as the initial and ongoing capacity-building training [73], poor network access and poor access to electricity [58,79], low financial investment [73], and unreliability or absence of infrastructure (eg, electricity and network) [80,81], hinder the implementation. As the skills and knowledge of HEWs vary from one to another, there should be mentoring and supportive supervision during the implementation. Studies showed that the inability to use the system could affect its implementation [26], and intensive training with continuous refreshment could help them realize the digitization of the community health information system [82].

### Limitations of the Study

The findings of this study should be interpreted in light of some limitations. Due to the nature of the study, which was cross-sectional, the inability to infer cause-and-effect relationships is present. As the study was focused on HEWs’ intention to use the eCHIS in a pilot district in Northwest Ethiopia, the sample size could affect the findings of this study, and covering larger areas at the regional and national levels is possible, including urban HEWs. Finally, the parcel approach used in this study may introduce parameter estimation bias.

### Conclusion

In conclusion, 39.3% (179/456) of HEWs scored above the mean of intention to use the eCHIS for community health data management and service provision. Factors associated with the intention to use the eCHIS were effort expectancy, self-expectancy, social influence, and hedonic motivation. The eCHIS has numerous advantages and a promising future in terms of improving data quality, use, and service delivery. Its adoption in the country, however, should focus on identifying all necessary prerequisites for successful implementation and advancing the community health information system. The implementation of the eCHIS should not skip factors that had no significant effect on intention to use the eCHIS, and further studies at the regional and national levels are recommended to investigate their correlation with intention to use the eCHIS. Model explainability was found in the study using factors that existed in the UTAUT2 model; however, it is recommended to examine the moderating effects of CHWs’ related variables to examine how the model constructs could influence HEWs’ intention to use the eCHIS.

## Abbreviations

CHW: community health worker
eCHIS: electronic community health information system
HEP: Health Extension Program
HEW: health extension worker
mHealth: mobile health
UTAUT: Unified Theory of Acceptance and Use of Technology
UTAUT2: Unified Theory of Acceptance and Use of Technology 2

## Data Availability

The data sets generated and analyzed during this study are available from the corresponding author on reasonable request.
